# Clinical Profile, Risk Factors, and Microbial Dysbiosis in Periodontitis: Findings from an Adult Cohort and Microbiome-Based Predictive Models

**DOI:** 10.3390/jcm15082994

**Published:** 2026-04-15

**Authors:** Sofia Jimenez de Nunzio, Jesus Pilo, Marta Bruna del Cojo, Caridad Margarita Arias-Macias, Barbara Manso de Gustin, Filipa Nunes, Eva Lago Pacheco, Clara Esteban Escobar, Francisco Tercero-Mora, Sergio Portal-Nuñez, Ana Adell Perez, Manuel Macias Gonzalez

**Affiliations:** 1 Spiral DNA Tech Corp, Calle Faraday 7, Fundacion Parque Cientifico de Madrid, 28049 Madrid, Spain; sofia.j@myspiralbox.com (S.J.d.N.); francisco.t@myspiralbox.com (F.T.-M.); 2 Departamento de Odontología, Facultad de Medicina, Universidad San Pablo-CEU, CEU Universities, Urbanización Montepríncipe, Boadilla del Monte, 28660 Madrid, Spain; marta.brunacojo@ceu.es (M.B.d.C.); cmargarita@ceu.es (C.M.A.-M.); barbara.mansogustin@ceu.es (B.M.d.G.); ana.adellperez@ceu.es (A.A.P.); 3 Instituto de Investigación Biomédica de Málaga y Plataforma en Nanomedicina—Ibima Plataforma Bionand, 29010 Malaga, Spain; jesuspilor@gmail.com; 4 Donte Group, Paseo del Club Deportivo 1., Edificio 3., 28223 Pozuelo de Alarcón, Spain; anunesp@dontegroup.com (F.N.); elago@dontegroup.com (E.L.P.); cesteban@dontegroup.com (C.E.E.); 5 Bone Physiopathology Laboratory, Instituto de Medicina Molecular Aplicada (IMMA), School of Medicine, Universidad San Pablo-CEU, CEU Universities, Urbanización Montepríncipe, Boadilla del Monte, 28660 Madrid, Spain; sergio.portalnunez@ceu.es; 6 Department of Basic Medical Sciences, School of Medicine, Universidad San Pablo-CEU, CEU Universities, Urbanización Montepríncipe, Boadilla del Monte, 28660 Madrid, Spain; 7 Unidad de Gestión Clínica de Endocrinología y Nutrición, Hospital Universitario Virgen de la Victoria, 29010 Malaga, Spain

**Keywords:** periodontitis, oral microbiome, dysbiosis, biomarkers, predictive model, malocclusion

## Abstract

**Background/Objective:** Periodontitis is a chronic inflammatory disease influenced by clinical, behavioral, and microbial determinants. However, the contribution of these factors to disease remains a topic of debate, particularly in untreated adult populations. This study aims to characterize the clinical, epidemiological, and microbial features associated with periodontitis in an adult cohort and to compare the discriminatory performance of microbiome-based predictive models with conventional clinical–behavioral models. **Methods:** A cross-sectional study was conducted in 943 adults. Periodontal status was determined by experienced clinicians according to the 2017 World Workshop on the Classification of Periodontal and Peri-implant Diseases and Conditions. Clinical variables, behavioral factors (smoking, bruxism, diet), intraoral conditions (caries and malocclusion), and systemic comorbidities were recorded. The oral microbiome was analyzed using targeted PCR for selected periodontal bacteria. Predictive models were constructed using logistic regression and least absolute shrinkage and selection operator (LASSO) variable selection. **Results:** Periodontitis was diagnosed in 47.2% of participants. Age, smoking, and bruxism were significantly associated with periodontitis. Malocclusion was the only significant intraoral predictor (OR = 2.00). Individuals with periodontitis exhibited increased levels of periodontopathogens, including *P. gingivalis*, *T. forsythia*, and *E. corrodens*, along with reduced levels of *S. mutans*. Microbiome-based models demonstrated superior discriminatory performance (AUC = 0.76, LASSO). *E. corrodens* and *C. sputigena* were independently associated with greater probing depth (*p* < 0.001). **Conclusions:** Microbiome-based predictive models, particularly at the species level, showed better discriminatory performance than conventional clinical–behavioral models. These findings support the potential utility of salivary microbial signatures as adjunctive, non-invasive biomarkers of periodontal inflammatory status.

## 1. Introduction

Periodontitis affects approximately 20–50% of the global population [1]. It is characterized by chronic inflammation caused by microbial infection, ultimately resulting in the destruction of the alveolar bone and, in some patients, tooth loss [2,3,4]. The microorganisms commonly associated with periodontal disease are typically anaerobic, Gram-negative bacteria such as *P. gingivalis*, *T. denticola*, *P. intermedia* (typically referred to as the periodontal Triad) [5,6,7], *A. actinomycetemcomitans* and *F. nucleatum* [8,9]. However, current evidence suggests that periodontitis is not solely driven by the overgrowth of these bacteria, but rather by a complex ecological change within the microbial community both in population and functionality [10,11,12].

Periodontitis is multifactorial and affected by other elements like salivary production [13], pH, abundance of nutrients and temperature [14], and impacted by genomics, diet, smoking, hygiene, oxygen exposure and antibiotic use [15,16,17], causing dysbiosis that leads to periodontal disease [18,19,20,21,22]. Another key factor in periodontal disease is the inflammatory response of the host. During periodontal infection, immune cells release proinflammatory cytokines—such as C-Reactive Protein, INF-γ, ll-1b, Il-8, Il-1α [23,24,25,26]. This, although aimed at controlling the infection, also produces toxic products, ultimately contributing to the destruction of periodontal tissue [27,28]. Chronic inflammation resulting from periodontal infection extends beyond the oral cavity. Numerous studies have established a strong association between periodontitis and the development or progression of systemic diseases [29] like colorectal cancer [30,31,32], cardiovascular disease [33,34,35], Alzheimer’s [36,37,38], Rheumatic arthritis [39,40,41], and diabetes [42,43,44].

Periodontal patients are reported to have a higher coronary risk than patients without periodontal disease. DNA of *P. gingivalis*, *A. actinomycetemcomitans*, *F. nucleatum* and others have been found in atheroma lesions [45]. The presence of these bacteria causes an inflammatory response that stimulates formation of atherosclerotic plaques by promoting the expression of cell adhesion molecules and diapedesis of monocytes in arterial walls. Additional hypotheses explain the potential role of bacteria promoting macrophage Low-Density Lipoprotein (LDL) uptake, crucial in the development of atherosclerotic plaques. The expression of inflammatory cytokines in the host response against periodontitis such as IL-1, IL-6, TNF-a and CRP are also commonly known to increase cardiovascular disease risk [33,45,46,47,48,49].

Despite the increase in oral microbiome research, there is still a strong focus on traditional models of periodontal infection based on the abundance of key pathogens such as *P. gingivalis* and *A. actinomycetemcomitans*. The role of non-classical bacterial species and their potential association with periodontal progression and systemic disease remains insufficiently explored.

Therefore, the aim of this study is to characterize the clinical, behavioral, and microbiological factors associated with periodontitis to determine the changes in the microbial profile. In addition, we sought to evaluate the potential role of specific bacterial species as markers of periodontal severity and their association with systemic comorbidities.

## 2. Materials and Methods

### 2.1. Study Design

A total of 943 adult participants were included in this cross-sectional study. Patients were consecutively recruited from approximately 400 dental clinics across Spain during routine clinical visits in which periodontal evaluation was indicated.

### 2.2. Sample Size

Sample size considerations were explored a priori using G*Power software (version 3.1.9.2), based on expected effect sizes and statistical parameters relevant to the study objectives.

### 2.3. Inclusion and Exclusion Criteria of Participants

All patients provided signed written consent, and their clinical data was collected by a dental professional treating them, including medical history, clinical dental findings and personal habits.

#### 2.3.1. Inclusion Criteria

- Patients older than 18 years old.
- Patients with at least 5 remaining teeth.
- Availability for periodontal examination and sample collection.

#### 2.3.2. Exclusion Criteria

- Antibiotic use 2 weeks prior to sample collection.
- Use of mouthwash 1 h prior to sample collection.
- Receiving any dental treatment prior to sample collection.
- Not signing the written consent form.
- Not having the dental exploration completed and shared by the dental professional.

No specific pre-operative procedures beyond standard clinical conditions were required prior to sample collection. The presence of minor blood contamination was not considered an exclusion criterion, as DNA quality and integrity were assessed after extraction using spectrophotometric analysis (NanoDrop) by A260/280 ratio, ensuring adequate purity of the genetic material for subsequent analyses.

### 2.4. Sample Collection and Clinical Data Collection

#### 2.4.1. Sample Collection

Gingival Crevicular Fluid (GCF) Samples were collected from around 400 Vitaldent dental clinics, spread throughout Spain. The recruitment period started on November 2023 and finished on 31 December 2024. The study was conducted in accordance with the Declaration of Helsinki and approved by the Ethics Committee of Universidad CEU San Pablo, protocol code 655/22/77, and date of 31 May 2023. Written informed consent was obtained from all participants prior to inclusion in the study.

Five Dentsply sterile paper tips were used to obtain Gingival Crevicular Fluid (GCF) from the patient, leaving each tip inside the periodontal pocket for 30 s. For teeth selection, dental professionals had to choose the deepest dental pocket for each quadrant, and the remaining tip was collected from a clinically relevant site, as determined by the dental professional.

Tips were then saved in a sterile microtube and stored at 4 °C.

#### 2.4.2. Clinical Data Collection

Clinical findings were described by trained dental professionals, following the diagnostic criteria established in the 2017 World Workshop on the Classification of Periodontal and Peri-implant Diseases and Conditions, based on clinical attachment loss (CAL), probing depth (PD), bleeding on probing (BOP), and radiographic bone loss. Clinical variables, behavioral factors (smoking, bruxism, diet), intraoral conditions (caries and malocclusion), and systemic comorbidities were also recorded by the trained clinicians.

### 2.5. DNA Extraction

Samples were inactivated at 70 °C for 20 min. DNA was extracted using MagMax DNA Multi-Sample Ultra 2.0 Kit (Thermo Fisher Scientific, Waltham, MA, USA), following the manufacturer’s instructions, using King Fisher purifying instrument (Thermo Fisher Scientific, Waltham, MA, USA). DNA concentration was measured using the Nanodrop 2000 Spectrophotometer (Thermo Fisher Scientific, Waltham, MA, USA), and those concentrations were later adjusted to 5 ng/µL for qPCR analysis.

### 2.6. qPCR Analysis and Quantification

Primers were designed by Isogen Life Science (De Meern, The Netherlands). SYBR Green Ref. 04887352001 from Roche (Basel, Switzerland) and Rox reference dye from Sigma-Aldrich (St. Louis, MO, USA) Ref. 4526-5mL.

Real-time Quantitative PCR (qPCR) analysis was performed on an ABI 7900HT Fast Real-Time PCR System (Applied Biosystems, Foster City, CA, USA) using 500 nM concentration of primers and the SYBR Green I enzyme and data were analyzed using Sequence Detection System software v2.4 (Applied Biosystems, Foster City, CA, USA).

Conditions for qPCR were standard (95 °C for 10 min; 40 cycles of 95 °C for 10 s; 60 °C for 30 s; and 72 °C for 15 s) and analyzed in the Sequence Detection System 2.4 software by Applied Biosystems. Samples, along with positive controls (10 pg of known concentrations of bacterial DNA pool per well) and a negative control (water) were analyzed for each of the targets.

Targets: 12 of the most common periodontal bacteria:

Aa: *Aggregatibacter actinomycetemcomitans;* Pg: *Porphyromonas gingivalis*; Tf: *Tannerella forsythia*; Pi: *Prevotella intermedia*; Fn: *Fusobacterium nucleatum*; Cr: *Campylobacter rectum*; En: *Eubacterium nodatum*; Ec: *Eikenella corrodens*; Cs: *Capnocytophaga sputigena*; Bm: *Bacteroides melaninogenicus*; Kn: *Klebsiella pneumoniae*; Sm: *Streptococcus mutans*.

Quantification of bacterial DNA was performed using standard curves generated from serial ten-fold dilutions of the bacterial DNA pool with known concentrations. Cycle threshold (Ct) values obtained from samples were interpolated against the corresponding standard curve to calculate the amount of bacterial DNA present in each sample. The results were expressed as picograms (pg) of bacterial DNA per reaction. All reactions were performed in duplicate, and melting curve analysis was conducted after amplification to confirm the specificity of the PCR products. Only reactions with amplification efficiencies between 90 and 110% and correlation coefficients (R^2^) greater than 0.98 were included in the analysis.

### 2.7. Statistical Analysis

All statistical analyses and figure generation were performed in RStudio R version 4.4.1 (2024-06-14 ucrt)—“Race for Your Life” (Copyright (C) 2024 The R Foundation for Statistical Computing Platform: x86_64-w64-mingw32/x64) using Quarto (v1.7.32) and packages from the tidyverse ecosystem. Data preprocessing, statistical testing, and visualization were conducted using standard packages from the tidy verse ecosystem, as well as specialized libraries for multivariate analysis. 

**Data preprocessing and normalization:** Clinical and demographic variables were encoded in categorical and continuous formats. Bacterial load data were quantified as absolute values by qPCR and Log10-transformed before visualization and multivariate analyses to reduce skewness and stabilize variance. Statistical tests comparing groups were always performed on raw, normalized (non-transformed) bacterial load values, whereas Log10-transformed values were used exclusively for plotting, dimensionality reduction, and regression modeling. For analysis involving multiple bacterial species or complexes, bacterial loads were either considered individually or were aggregated into predefined microbial complexes by summing the abundances within each complex.

**Descriptive analyses:** Descriptive statistics were used to summarize the demographic, systemic, behavioral, and intraoral characteristics of the study population. Categorical variables were reported as proportions with 95% confidence intervals, while continuous variables were summarized as means with standard deviations or medians with interquartile ranges, according to their distribution. These analyses were purely descriptive, and no inferential statistical tests were applied.

**Correlation analysis:** Pairwise associations between behavioral factors, systemic health antecedents, and intraoral variables were assessed using Spearman’s rank correlation coefficient (ρ). The statistical significance of the correlation coefficients was evaluated using two-sided hypothesis tests. When multiple correlations were tested simultaneously, *p*-values were adjusted for multiple comparisons using the Benjamini–Hochberg false discovery rate (FDR) correction, as appropriate.

**Group comparisons:** Between-group comparisons for categorical variables were performed using Pearson’s χ^2^ test or Fisher’s exact test. Trends across ordered age categories were evaluated using the Cochran–Armitage trend test.

Given the skewed distribution of microbial load data, normality and data distribution was assessed using the Shapiro–Wilk test prior to statistical analysis. As several microbial load variables exhibited non-normal distributions, appropriate statistical methods were selected accordingly, including non-parametric tests when required. Log10 transformation was applied exclusively for visualization purposes to improve interpretability of the figures, while all univariate statistical analyses were conducted using the original normalized values (adjusted by total DNA content). For multivariate analyses, such as principal component analysis (PCA), Log10 (value + offset) transformation followed by scaling was applied to stabilize variance and account for the wide dynamic range of microbial loads.

Depending on distributional assumptions, comparisons between two groups were conducted using either Student’s *t*-test or the Wilcoxon rank-sum test.

**Regression modeling:** Logistic regression models were used to evaluate associations between clinical, behavioral, and microbiological variables and binary outcomes (example: gingivitis or periodontitis). Models were adjusted for relevant covariates, including age, smoking habit, hypertension, caries history, bruxism, and malocclusion, as appropriate. For continuous outcomes, such as periodontal depth, linear regression models were fitted, with age modeled per 10-year increment. Model assumptions were verified by inspecting residuals and variance inflation factors. To handle multicollinearity and variable selection in models including multiple bacterial predictors, penalized regression using the least absolute shrinkage and selection operator (LASSO) was applied. Optimal penalty parameters were selected by 10-fold cross-validation.

**Dimensionality reduction:** Principal component analysis (PCA) was performed on Log10-transformed bacterial load data to explore multivariate patterns and group separation. PCA results were visualized using biplots, with loadings used to identify the bacterial species contributing most strongly to each principal component.

**Model performance and validation:** Model discrimination was evaluated using receiver operating characteristic (ROC) curves, and performance was quantified by the area under the curve (AUC) with 95% confidence intervals. Comparisons between ROC curves were conducted using the DeLong test.

**Statistical significance:** All statistical tests were two-sided. An FDR or *p*-value < 0.05 was considered statistically significant. When applicable, multiple testing corrections were applied. Statistical significance is denoted in figures using asterisks (* *p* < 0.05, ** *p* < 0.01, *** *p* < 0.001).

## 3. Results

### 3.1. Distribution of Subject Population

**Figure 1:**
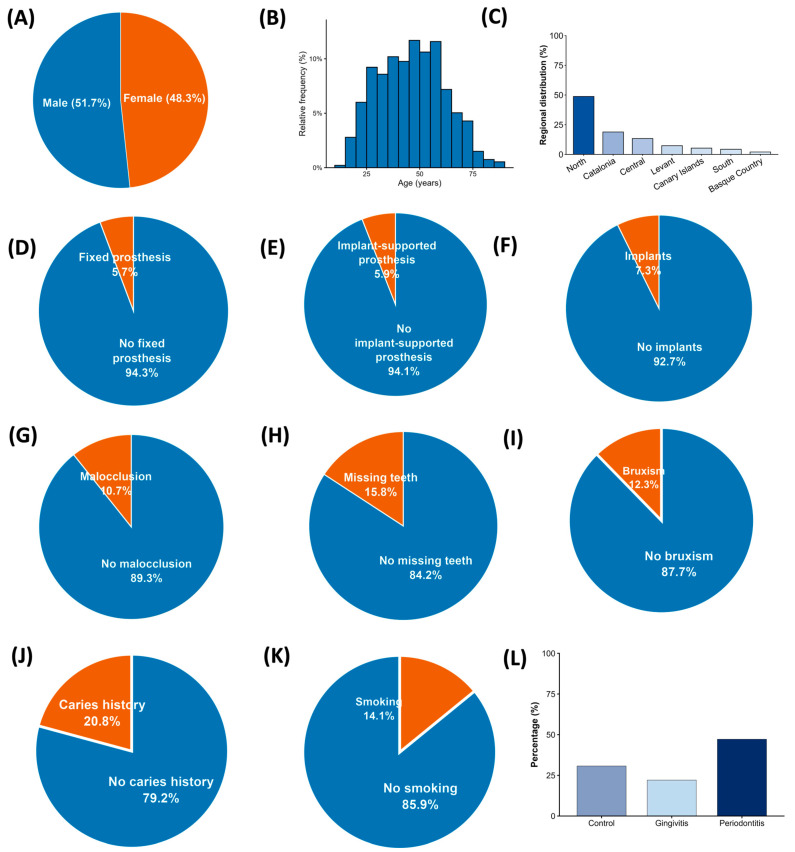
General characteristics of the study population. (**A**) Sex distribution. (**B**) Age distribution of patients in years. (**C**) Regional distribution of participants. (**D**–**J**) Clinical and intraoral characteristic prevalence in whole population: presence of fixed prosthesis, implant-supported prosthesis, implants, malocclusion, missing teeth, orthodontic appliance, removable prosthesis, soft-tissue lesions, bruxism, caries history, diabetes, and hypertension. (**K**) Smoking habits. (**L**) Periodontal status distribution (control, gingivitis, and periodontitis). All graphs represent descriptive data only; no statistical tests were applied. Among all participants (n = 943), 30.7% were controls, 22.1% gingivitis, and 47.2% periodontitis, showing that almost half of the population presented periodontitis.

The study population consisted of 943 patients recruited in 400 Vitaldent clinics, distributed all over Spain’s geography; participants were distributed across different age groups, with different stages of periodontal disease. (Figure 1A) In total, 48.3% were female and 51.7% were male.

Most of the patients (Figure 1B) were from the ages of 31–50 (39.8%) and 51–70 (34%) meaning that the study is mostly shaped by middle-aged and older adults (Figure 1D–J).

Almost half of the study population is formed by individuals from the Northern regions of Spain, representing 48.8% of the patients, followed by the Catalonia region with 18.9%, the Center region with 13.4%, the Levant region, representing only 7.3% of the study population, The Canary Islands, making up 5% of the sample, and lastly Andalucia with 4.3% and Basque country with 2%.

Most of the patients did not have a fixed prosthesis, an implant-supported prothesis, implants or malocclusion. In this cohort (Figure 1K), 12.3% have bruxism, 15.8% are described as having missing teeth and 20.8% have cavities, this being the most reported intraoral finding by dentists that does not fall into periodontal disease categories. Interestingly, only 14.1% of patients are reported to be smokers.

Finally, the analysis of periodontal status is as follows: Almost half of participants (47.2%) have periodontal disease. Only 22.1% are described as having gingivitis and 30.7% are described as having no periodontal pathology. Altogether, the descriptive data reflect an adult population with a high prevalence of periodontal disease.

### 3.2. Smoking and Age, as Well as Clinical Findings Like Bruxism and Cavities, Are Associated with an Increased Likelihood of Periodontitis and Gingivitis

**Figure 2:**
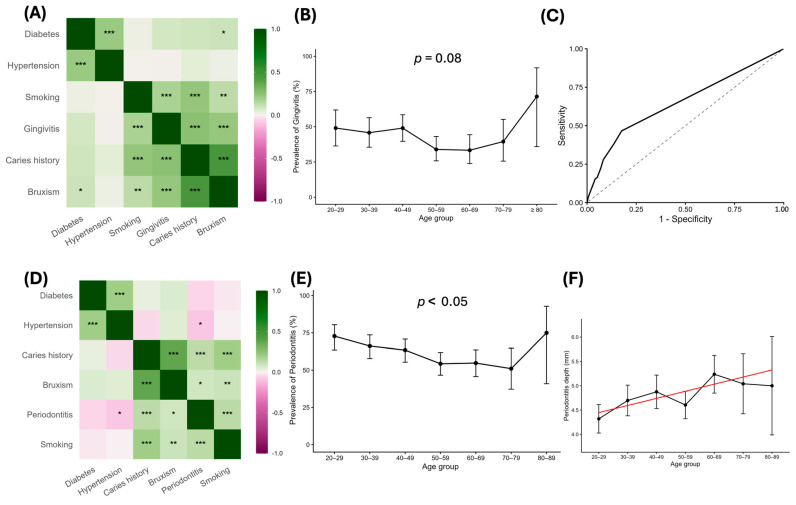
**Relationships between systemic, behavioral, and demographic factors in gingivitis and periodontitis.** (**A**) Correlation heatmap among gingivitis patients and personal-history incidences (diabetes, hypertension, smoking, caries history, bruxism). Color indicates correlation coefficients. (**B**) Prevalence of gingivitis by age. Patients were grouped by decades (20–29, 30–39, …, ≥80 years). Error bars represent 95% confidence intervals for proportions. (**C**) ROC curve obtained from a logistic regression model including smoking habits, bruxism, and caries history as predictors of gingivitis. The solid line represents the true positive rate (sensitivity) versus the false positive rate (1—specificity). The curve represents the model’s discriminative performance relative to the 45° reference line. (**D**) Correlation heatmap among periodontitis patients and personal-history incidences (diabetes, hypertension, smoking, caries history, bruxism). Color shows correlation coefficients. (**E**) Prevalence of periodontitis by age. Patients were grouped by decades (20–29, 30–39, …, 80–89 years). Error bars represent 95% confidence intervals for proportions. (**F**) Periodontitis depth tendency by age. Y-axis shows the mean of periodontitis depth (mm) in each 10-year age group; black points represent means with 95% CIs and the black line connects group means. The red line is the adjusted trend from a linear model depth ~age/10 + smoking + bruxism + caries history + hypertension. Adjusted age effect: +0.15 mm per 10 years (95% CI 0.05–0.24; *p* = 0.0018; R^2^ of the adjusted model). *p*-values come from χ^2^ tests, Fisher’s exact tests, Cochran–Armitage trend tests, and linear regression models as appropriate. Asterisks denote significance * *p* < 0.05, ** *p* < 0.01, *** *p* < 0.001.

We found a positive correlation (Figure 2A) between gingivitis and smoking, as well as a significant relationship between clinical oral findings like bruxism and cavities (Figure 2B). No statistical significance was detected between ascending age and the likelihood of having gingivitis. It is worth noting that patients older than 60 years represent a higher stratum of the gingival population, which suggests a cumulative effect over time and prolonged exposure to risk factors. While all these variables are associated with increased likelihood of having gingivitis (Figure 2C), they are not on their own enough to predict it as shown in the regression model. It presented a moderate discriminative capacity, with high specificity but low sensitivity, meaning that the model is more efficient at identifying patients without gingivitis than patients with gingivitis. Similar results were observed with periodontitis (Figure 2D). The heatmap shows a positive correlation between periodontitis and smoking, bruxism, and cavities, and unlike gingivitis, there is also a positive correlation between (Figure 2D) hypertension and periodontitis. This time (Figure 2E), there is a statistical significance regarding a general ascending trend with age and periodontal disease, although no linear pattern is characterized, with a decrease in patients aged between 40 and 70 and an upturn between ages 70–89. This variation could be attributed to dental loss or exposure to periodontal treatment between the 40–70 age range, especially if we consider that most of the subject population is precisely between this age group and they are patients already in dental clinics. This sudden increase could be attributed to the cumulative effects of tissue damage, chronic inflammation and comorbidities associated with aging. These findings support an association between age and periodontal disease (Figure 2F). There is a strong statistical significance between pocket depth (a critical parameter to track periodontal progression) and age, even after adjusting the model with smoking, bruxism, cavities and hypertension. This result indicates that pocket depth intensifies with age, and the influence of age is independent of other risk factors.

### 3.3. Malocclusion Is a Relevant Intraoral Finding Related to Periodontitis

**Figure 3:**
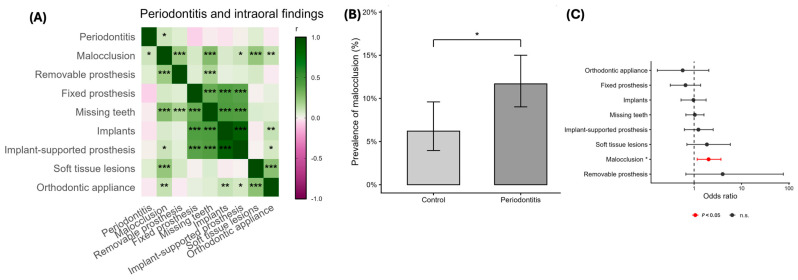
**Relationships between intraoral conditions and periodontitis.** (**A**) Correlation heatmap among intraoral clinical variables and periodontitis (malocclusion, removable and fixed prostheses, missing teeth, implants, implant-supported prostheses, soft-tissue lesions, and orthodontic appliances). Color represents correlation coefficients, and asterisks indicate statistical significance (* *p* < 0.05, ** *p* < 0.01, *** *p* < 0.001). (**B**) Prevalence of malocclusion in control subjects and patients with periodontitis. Bars represent proportions with 95% confidence intervals. (**C**) Age-adjusted associations between intraoral findings and periodontitis. Forest plot showing odds ratios (Log10 scale) with 95% confidence intervals from logistic regression models adjusted for age. Red markers indicate variables with *p* < 0.05 after age adjustment.

Among the clinical findings of malocclusion, removable prosthesis, fixed prosthesis, missing teeth, implants, implant-supported prosthesis, soft-tissue lesions and orthodontic appliances, only malocclusion (Figure 3A) was found to have a significant correlation with periodontal disease. These findings, compared to the previous in Figure 2, seem to have a more limited role in explaining periodontal disease. The prevalence of malocclusion is significantly higher in periodontal patients compared to controls (Figure 3B). Additionally, the multivariant analysis adjusted by age (Figure 3C) confirms that only malocclusion has a significant and independent association with periodontal disease. Altogether, the results seem to indicate that clinical findings like removable prothesis, fixed prothesis, missing teeth, implants, implant-supported prothesis, soft-tissue lesions and orthodontics appliances have a lower predictive capability regarding periodontal disease, with malocclusion being the only factor consistently associated with periodontitis.

### 3.4. Bacterial Composition Differs Significantly Between Periodontal Patients and Controls

**Figure 4:**
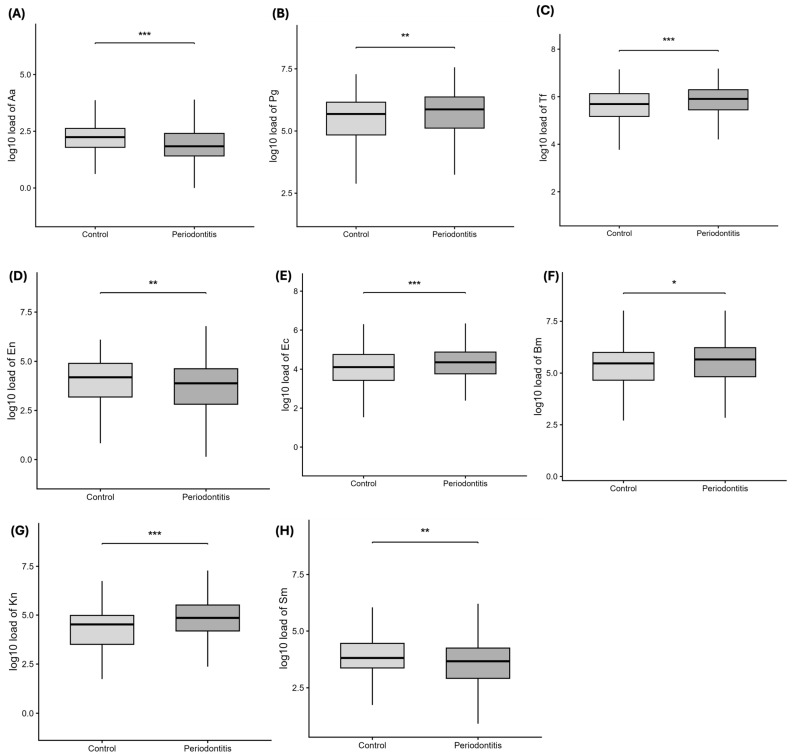
**Bacterial composition in periodontal disease.** Boxplots showing bacterial loads (Log10 scale) in control and periodontitis groups. Boxes represent medians and interquartile ranges; whiskers follow Tukey’s rule. (**A**) *Aggregatibacter actinomycetemcomitans*, (**B**) *Porphyromonas gingivalis*, (**C**) *Tannerella forsythia*, (**D**) *Eubacterium nodatum*, (**E**) *Eikenella corrodens*, (**F**) *Bacteroides melaninogenicus*, (**G**) *Klebsiella* sp. (**H**) *Streptococcus mutans*. Statistical comparisons (Wilcoxon or Student’s *t*-test) were performed on raw data; values are plotted as Log10-transformed loads. Asterisks denote statistical significance (* *p* < 0.05, ** *p* < 0.01, *** *p* < 0.001).

Bacterial quantification in patients showed a significant change in oral composition between periodontal patients and controls. Interestingly, bacteria commonly associated with periodontal disease like *Aggregatibacter actinomycetemcomitans* (Figure 4A) were reported to be higher in control groups. A higher abundance of Gram-negative bacteria like (Figure 4B) *Porphyromonas gingivalis*, (Figure 4C) *Tannerella forsythia*, (Figure 4D) *Eikenella corrodens*, (Figure 4E) *Bacteroides melaninogenicus*, and (Figure 4G) *Klebsiella* sp. was found. Other bacteria found higher in control groups are (Figure 4H) *Streptococcus mutans* (Sm) and (Figure 4E) *Eubacterium nodatum*. Species-specific odds ratios derived from the bacteria-only model are presented in Appendix A Table A1.

### 3.5. Periodontal Disease Is Associated with Patterns Consistent with Differences in Bacterial Abundance Within the Analyzed Bacterial Panel

**Figure 5:**
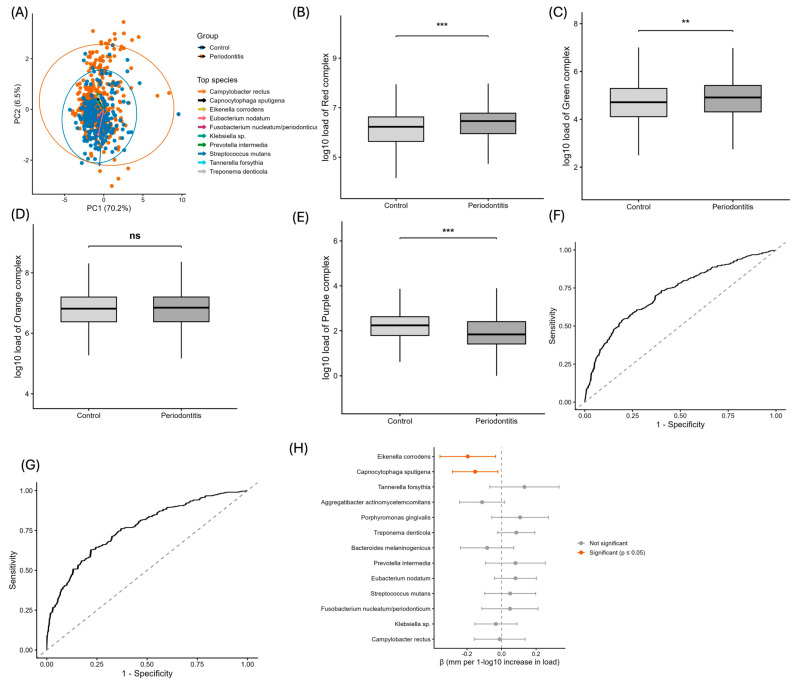
**Bacterial complex analysis and predictive models in periodontitis** (**A**) Principal component analysis (PCA) based on bacterial load (Log10). Ellipses represent 95% confidence intervals for each group, and labeled points indicate the top contributing species. Rows show the positive direction of each principal component and the species-loading vectors, with arrow length proportional to their contribution to PC1/PC2. (**B**–**E**) Boxplots of bacterial complexes comparing controls and periodontitis, showing bacterial load on a Log10 scale. (**B**) Red complex (*Porphyromonas gingivalis*, *Tannerella forsythia*, *Treponema denticola*), (**C**) green complex (*Eikenella corrodens*, *Capnocytophaga sputigena*), (**D**) orange complex (*Prevotella intermedia*, *Fusobacterium*, *Campylobacter rectus*, *Eubacterium nodatum*), and (**E**) purple complex (*Aggregatibacter actinomycetemcomitans*). (**F**,**G**) Receiver operating characteristic (ROC) curves for adjusted models predicting periodontitis: (**F**) complex-based model (AUC = 0.721, 95% CI 0.684–0.758) and (**G**) species-based model (AUC = 0.764, 95% CI 0.724–0.803). (**H**) Forest plot of regression coefficients (β) showing the association between individual bacterial loads and periodontitis depth (mm), adjusted for age, smoking, hypertension, caries history, and malocclusion. Horizontal lines represent 95% confidence intervals. Orange indicates species significantly associated with increased depth (*p* < 0.05). Between-group comparisons were assessed using Wilcoxon or Student’s *t*-tests according to distributional assumptions. Multivariate associations were evaluated by logistic regression and penalized LASSO models, adjusted for relevant covariates (age, smoking, hypertension, caries history, and malocclusion). Model discrimination was assessed by area under the ROC curve (AUC) with 95% confidence intervals, and model stability by 10-fold cross-validation. Asterisks denote statistical significance (** *p* < 0.01, *** *p* < 0.001).

Analysis of bacterial complexes in periodontitis vs the control group revealed that (Figure 5A) while all bacteria in the study can be found in both periodontal patients and the control group, the significant difference lies in the relative abundance in which they are quantified. Periodontal patients have differences in relative abundance patterns across selected bacterial species (Figure 5A), rather than a purely taxonomic dysbiosis. Additionally, a higher dispersion observed in periodontal patients suggests an increase in microbial heterogeneity.

Red and green complex bacteria showed a significant increase in periodontal patient’s vs controls. Remarkably, *Aggregibacter actinomycetemcomitans*, a bacterium commonly associated with periodontitis, showed a significant decrease. This pattern (Figure 5B–E) seems to indicate an alteration in oral microbiome, with an increase in the red and green complex bacteria.

Specific differences (Figure 5F) in relative abundance between species better capture dysbiosis than the analysis grouped by complexes. Furthermore, both models demonstrated good internal stability after cross-validation (10-fold CV), reinforcing the consistency of the results and the possibility of replicating them in other cohorts, as well as using these bacteria as potential markers of the disease.

Another interesting finding was that two species (Figure 5G,H), *Eikenella corrodens* and *Capnocytophaga sputigena*, are highly associated with pocket depth, an important clinical finding used as a marker of periodontal progression. Even after adjusting the statistical model with age, smoking, hypertension, cavities and malocclusion, the findings remained unaltered.

### 3.6. Eikenella corrodens (Ec) Is More Abundant in Patients with Cardiovascular Disease

**Figure 6:**
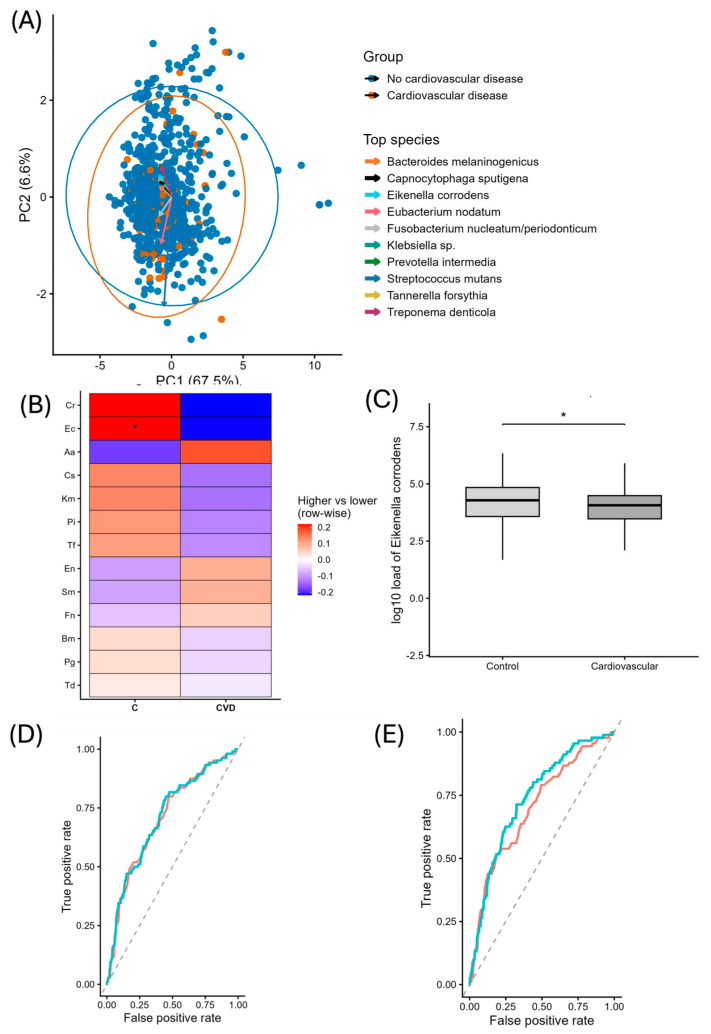
**Bacterial complex analysis and predictive models in periodontitis.** (**A**) Principal component analysis (PCA) based on bacterial load (Log10). Ellipses represent 95% confidence intervals for each group, and labeled points indicate the top contributing species. Arrows show the positive direction of each principal component and the species-loading vectors, with arrow length proportional to their contribution to PC1/PC2. (N (NCD = 83; N(CVD) = 860.) (**B**) Heatmap showing differences in analyzed bacterial abundance between control (**C**) and cardiovascular disease (CVD) groups. For each bacterium (row), colors indicate relative abundance between groups using Log10-transformed values with offset; red denotes higher median abundance in that group and blue denotes lower abundance. Asterisks indicate statistically significant differences between groups based on the Wilcoxon test. (**C**) Comparison of *Eikenella corrodens* abundance between control and cardiovascular groups. Bacterial load was compared using the Wilcoxon rank-sum test. (* *p* < 0.05) (**D**) Receiver operating characteristic (ROC) curves comparing a clinical model based on age and smoking (AUC = 0.708) with an extended model including age, smoking, and *Eikenella corrodens* abundance (AUC = 0.711). The diagonal dashed line represents the performance of a non-informative classifier. The DeLong test did not show statistically significant differences (*p* = 0.475). (**E**) Receiver operating characteristic (ROC) curves comparing a clinical model based on age and smoking (AUC = 0.708) with an extended model including age, smoking, and the full panel of all bacterial abundances (AUC = 0.740). The diagonal dashed line represents the performance of a non-informative classifier. The inclusion of bacterial variables resulted in a slight increase in discriminative performance (ΔAUC = 0.038). DeLong test did not show statistically significance differences (*p* = 0.068). The pink line represents the clinical model (age and smoking), while the turquoise line represents the extended model including bacterial variables. The dashed gray line indicates the performance of a non-informative classifier.

Microbiota profiling in patients revealed a higher presence of *Eikenella corrodens* in periodontal patients with reported cardiovascular disease (CVD) such as hypertension, heart attack, coronary insufficiency, among others, compared to periodontal patients without CVD (Figure 6A). Although bacteria related to periodontal disease and cardiovascular disease like *Porphyromonas gingivalis* is also more abundant, it does not show a statistical significance (Figure 6B), unlike Ec, which was statistically significantly found more abundant in CVD patients (Figure 6C). When adding other factors to the statistical model, the results showed that although Ec is more abundant in CVD, it did not retain independent predictive value after multivariant adjustment (Figure 6D,E). Full multivariable regression results are provided in Appendix A Table A2. Other systemic variables did not show statistically significant associations.

## 4. Discussion

Periodontitis is one of the most prominent dental diseases worldwide, and its complexity along with its connection to systemic diseases makes it necessary to understand and identify predictive markers to improve prevention and treatment strategies. The findings in this work support the idea that periodontal disease is a multifactorial pathology; factors like age, smoking and other comorbidities like bruxism, cavities or malocclusion are significantly associated with periodontitis. These findings are consistent with previous studies reporting age and smoking as major risk factors for periodontitis [50,51]. Although cavities were associated with periodontitis, the higher abundance of *Streptococcus mutans* in control individuals suggests that cavities may reflect overall oral health status rather than acting as a direct microbial driver of periodontal disease.

Beyond traditional periodontal pathogens (as *P. gingivalis*, *T. denticola*, *P. intermedia*, *A. actinomycetemcomitans* and *F. nucleatum*), other species such as *Eikenella corrodens* and *Capnocytophaga sputigena* were associated with increased periodontal pocket depth, suggesting that they may reflect greater disease severity or advanced disease progression. However, given the cross-sectional nature of the study, no temporal relationship can be established, and these findings are not evidence of a causal role in progression. This supports emerging evidence suggesting that non-classical bacterial species may play a relevant role in periodontal dysbiosis [52,53]. Although their exact biological contribution remains unclear, these microorganisms may act either as active contributors to tissue destruction or as ecological markers of advanced periodontal dysbiosis. To establish a clear role, longitudinal studies need to be conducted. This highlights the need to broaden the current pathogen-centered view of periodontal disease toward a more ecological perspective [54].

*Eikenella corrodens* was more abundant in patients with CVD compared to control groups highlighting the potential relevance of non-classical bacterial species beyond periodontal disease. However, this association did not remain significant after adjustment, suggesting that non-classical bacteria like *E. corrodens* may reflect advanced inflammatory states or higher inflammatory burden in a periodontal context rather than acting as direct drivers of systemic disease. Similar associations between periodontal inflammation and cardiovascular disease have been widely reported [55], although the role of specific bacterial species remains unclear.

The association between *E. corrodens* and increased periodontal pocket depth further supports the hypothesis that this species may be linked to later stages of periodontal disease. In this context, the relationship between periodontitis and CVD could be mediated more by disease severity and cumulative inflammatory exposure than by the presence of specific bacterial profiles alone. This could also explain why traditional periodontal bacteria were not statistically significant in CVD. Overall, these findings suggest that systemic associations may be more strongly related to the host inflammatory response and disease severity than to individual bacterial taxa. Further studies, including the evaluation of inflammatory biomarkers, are needed to better understand these relationships.

This paper has some limitations that should be considered. Due to the cross-sectional design of the study, a temporal relationship between microbial dysbiosis and periodontal disease progression cannot be established. Therefore, the observed microbial patterns should be interpreted as associations with disease status or severity at the time the sample was taken and not considered as evidence of causative mechanisms or progression pathways. To establish the latter claim, longitudinal studies are needed. Secondly, the use of targeted qPCR limits the analysis to a predefined set of bacterial species; other microorganisms not included in the panel of study, that could be of potential interest, are being excluded. Therefore, the observed patterns should be interpreted as changes in the relative abundance of the selected bacterial panel rather than a representation of the full complexity of the oral microbial community. A more complete characterization would require untargeted approaches, such as 16S rRNA sequencing. However, it is important to note that qPCR was intentionally selected for this study due to its greater applicability in clinical settings. Compared to sequencing-based methods, qPCR is more cost effective, faster and more accessible, making it a practical tool for both dental professionals and patients to obtain clinically relevant information. Although the multi-center design is a strength, it also plays a role in the heterogeneity of diagnosis by different professionals. Dental professionals who participated in this study received standardized training implemented by the same person to ensure that sample collection and clinical findings were noted using the same classification method. However, we recognize that some inter-examiner variability could remain. Lastly, because the study is being done in dental patients in dental clinics, this could bias and limit the generalizability of the conclusions to the overall population. Despite these limitations, we strongly believe that the large and standardized sample size, based on the population of 400 clinics across Spain, and the continuous training provided monthly to all the clinics and dental professionals in the study to homogenize criteria in periodontal diagnosis and sample collection, provide robust evidence to support the findings in this paper.

## 5. Conclusions

In conclusion, the study provides an integrated vision of periodontal pathology, showing a strong link between smoking, age and oral microbiome. The analyzed bacterial panel presents an identifiable pattern marked by a higher presence of red and green complex bacteria, as well *Eikenella corrodens* and *Capnocytophaga sputigena*, as emerging independent markers of advanced periodontal disease. An additional observation was the statistically significant abundance of Ec in CVD patients opening the path to further works to describe the role of non-classical bacteria in the relationship between periodontal disease and systemic diseases, although it does not appear to act as an independent variable. Given that oral microbiota can be tested and measured using qPCR analysis, there is strong evidence to support that oral microbiota could be used as a base to improve diagnosis models, preventative strategies, and clinical biomarkers for periodontal disease. We support that these findings have major implications for periodontal medicine, as well as systemic medicine. Overall, these findings support a shift toward a more ecological and integrative understanding of periodontal disease.

## Data Availability

The datasets generated and analyzed during the current study are not publicly available due to patient privacy and ethical restrictions but are available from the corresponding author on reasonable request.

## Abbreviations

Aa/*A. actinomycetemcomitans*: *Aggregatibacter actinomycetemcomitans*; Pg/*P. gingivalis*: *Porphyromonas gingivalis*; Tf/*T. forsythia*: *Tannerella forsythia*; Pi/*P. intermedia*: *Prevotella intermedia*; Fn/*F. nucleatum*: *Fusobacterium nucleatum;* Ec/*E. corrodens*: *Eikenella* corrodens; Cs/*C. sputigena*: *Capnocytophaga sputigena*; Sm/*S. mutans*: *Streptococcus mutans*; Bm: *Bacteroides melaninogenicus*; Cr: *Campylobacter rectum;* En: *Eubacterium nodatum*; Kn: *Klebsiella pneumoniae*; Td/*T. denticola*: *Treponema denticola*; Ks/*Klebsiella* sp.: *Klebsiella species*; LDL: Low-Density Lipoprotein; PCR: Polymerase Chain Reaction; qPCR: Quantitative Polymerase Chain Reaction; DNA: Deoxyribonucleic Acid; ABI: Applied Biosystems; CGF: Crevicular Gingival Fluid; CRP: C-Reactive Protein; IFN-γ: Interferon gamma; IL-1β: Interleukin-1 beta; L-6: Interleukin-6; IL-8: Interleukin-8; TNF-α: Tumor Necrosis Factor alpha; AMI: Acute Myocardial Infarction.

## Appendix A

### Appendix A.1. Odds Ratios (ORs) and 95% Confidence Intervals (95% CI) from Logistic Regression Models Adjusted for Age and Smoking, in Which Each Bacterial Species Was Analyzed Separately as a Predictor of Cardiovascular Disease

Bacterial loads were modeled as Log10-transformed continuous variables. ORs represent the change in odds of cardiovascular disease per one-unit increase in Log10 bacterial load, adjusted for age and smoking status. N indicates the number of individuals included in each model after complete-case filtering.

**Table A1:** Bacteria OR multivariable model (all bacteria).

Model	Bacteria	Term	OR	CI95_Low	CI95_High	*p*_Value	N
Multivariable (all bacteria only)	*Aggregatibacter actinomycetemcomitans*	*Aggregatibacter actinomycetemcomitans* (Log10)	1.2126501	0.967502583	1.50931625	0.088234025	726
Multivariable (all bacteria only)	*Bacteroides melaninogenicus*	*Bacteroides melaninogenicus* (Log10)	1.012117174	0.745478079	1.386935396	0.939291304	726
Multivariable (all bacteria only)	*Campylobacter rectus*	*Campylobacter rectus* (Log10)	0.930637663	0.685631211	1.317655379	0.663379692	726
Multivariable (all bacteria only)	*Capnocytophaga sputigena*	*Capnocytophaga sputigena* (Log10)	0.93387083	0.689899114	1.27098357	0.660058363	726
Multivariable (all bacteria only)	*Eikenella corrodens*	*Eikenella corrodens* (Log10)	0.786133156	0.566835134	1.105183066	0.156605784	726
Multivariable (all bacteria only)	*Eubacterium nodatum*	*Eubacterium nodatum* (Log10)	1.13599572	0.891369468	1.477493225	0.321480721	726
Multivariable (all bacteria only)	*Fusobacterium nucleatum/periodonticum*	*Fusobacterium nucleatum/periodonticum* (Log10)	2.233954032	1.251194879	4.160220343	0.00886866	726
Multivariable (all bacteria only)	*Klebsiella* sp.	*Klebsiella* sp. (Log10)	0.849336741	0.646888877	1.120035081	0.242224854	726
Multivariable (all bacteria only)	*Porphyromonas gingivalis*	*Porphyromonas gingivalis* (Log10)	1.015274529	0.694843608	1.52509816	0.939693692	726
Multivariable (all bacteria only)	*Prevotella intermedia*	*Prevotella intermedia* (Log10)	0.8122881	0.527347878	1.262590247	0.34959497	726
Multivariable (all bacteria only)	*Streptococcus mutans*	*Streptococcus mutans* (Log10)	1.175745685	0.945425378	1.462901122	0.145239274	726
Multivariable (all bacteria only)	*Tannerella forsythia*	*Tannerella forsythia* (Log10)	0.795181137	0.445611693	1.415894742	0.436152056	726
Multivariable (all bacteria only)	*Treponema denticola*	*Treponema denticola* (Log10)	0.936232027	0.786941928	1.121800572	0.464869253	726

### Appendix A.2. Odds Ratios (ORs) and 95% Confidence Intervals (95% CI) from a Multivariable Logistic Regression Model Including All Bacterial Species Simultaneously, Adjusted for Age and Smoking Status

Bacterial loads were modeled as Log10-transformed continuous variables. The outcome was cardiovascular disease. ORs represent the association between each bacterial species and cardiovascular disease mutually adjusted for all other bacteria in the model and for clinical covariates. N indicates the number of individuals included in the analysis after complete-case filtering.

**Table A2:** Bacteria OR multivariable model (all bacteria + age + smoking).

Model	Bacteria	Term	OR	CI95_Low	CI95_High	*p*_Value	N
Multivariable (all bacteria + age + smoking)	*Aggregatibacter actinomycetemcomitans*	*Aggregatibacter actinomycetemcomitans* (Log10)	1.254658421	0.997512034	1.568932501	0.048752117	718
Multivariable (all bacteria + age + smoking)	*Bacteroides melaninogenicus*	*Bacteroides melaninogenicus* (Log10)	0.936818303	0.68707351	1.286708759	0.682833817	718
Multivariable (all bacteria + age + smoking)	*Campylobacter rectus*	*Campylobacter rectus* (Log10)	1.047408845	0.753894764	1.525273535	0.795049825	718
Multivariable (all bacteria + age + smoking)	*Capnocytophaga sputigena*	*Capnocytophaga sputigena* (Log10)	0.970625561	0.711481281	1.332547536	0.851908941	718
Multivariable (all bacteria + age + smoking)	*Eikenella corrodens*	*Eikenella corrodens* (Log10)	0.779863359	0.551267877	1.117787866	0.167390786	718
Multivariable (all bacteria + age + smoking)	*Eubacterium nodatum*	*Eubacterium nodatum* (Log10)	1.099815126	0.850198562	1.446735758	0.482087888	718
Multivariable (all bacteria + age + smoking)	*Fusobacterium nucleatum/periodonticum*	*Fusobacterium nucleatum/periodonticum* (Log10)	1.77321246	0.98551701	3.372364283	0.069183481	718
Multivariable (all bacteria + age + smoking)	*Klebsiella* sp.	*Klebsiella* sp. (Log10)	0.808957725	0.602832506	1.091224282	0.160348265	718
Multivariable (all bacteria + age + smoking)	*Porphyromonas gingivalis*	*Porphyromonas gingivalis* (Log10)	0.999772536	0.674747379	1.526567143	0.999127747	718
Multivariable (all bacteria + age + smoking)	*Prevotella intermedia*	*Prevotella intermedia* (Log10)	0.92233659	0.58442171	1.472195752	0.731152935	718
Multivariable (all bacteria + age + smoking)	*Streptococcus mutans*	*Streptococcus mutans* (Log10)	1.140958075	0.910604581	1.432909795	0.253230736	718
Multivariable (all bacteria + age + smoking)	*Tannerella forsythia*	*Tannerella forsythia* (Log10)	0.786805364	0.429674379	1.428577613	0.433073662	718
Multivariable (all bacteria + age + smoking)	*Treponema denticola*	*Treponema denticola* (Log10)	1.023807532	0.853101357	1.237790346	0.803816178	718
